# MicroRNA-182 targets SMAD7 to potentiate TGFβ-induced epithelial-mesenchymal transition and metastasis of cancer cells

**DOI:** 10.1038/ncomms13884

**Published:** 2016-12-20

**Authors:** Jingyi Yu, Rong Lei, Xueqian Zhuang, Xiaoxun Li, Gang Li, Sima Lev, Miguel F. Segura, Xue Zhang, Guohong Hu

**Affiliations:** 1 The Key Laboratory of Stem Cell Biology, Institute of Health Sciences, Shanghai Institutes for Biological Sciences, Chinese Academy of Sciences & Shanghai Jiao Tong University School of Medicine, University of Chinese Academy of Sciences, Shanghai 200031, China; 2 Molecular Cell Biology Department, Weizmann Institute of Science, Rehovot 76100, Israel; 3 Department of Pathology, New York University School of Medicine, New York, New York 10016, USA

## Abstract

The transforming growth factor β (TGFβ) pathway plays critical roles during cancer cell epithelial-mesenchymal transition (EMT) and metastasis. *SMAD7* is both a transcriptional target and a negative regulator of TGFβ signalling, thus mediating a negative feedback loop that may potentially restrain TGFβ responses of cancer cells. Here, however, we show that TGFβ treatment induces *SMAD7* transcription but not its protein level in a panel of cancer cells. Mechanistic studies reveal that TGFβ activates the expression of microRNA-182 (miR-182), which suppresses SMAD7 protein. miR-182 silencing leads to SMAD7 upregulation on TGFβ treatment and prevents TGFβ-induced EMT and invasion of cancer cells. Overexpression of miR-182 promotes breast tumour invasion and TGFβ-induced osteoclastogenesis for bone metastasis. Furthermore, miR-182 expression inversely correlates with SMAD7 protein in human tumour samples. Therefore, our data reveal the miR-182-mediated disruption of TGFβ self-restraint and provide a mechanism to explain the unleashed TGFβ responses in metastatic cancer cells.

## Abstract (web summary)

SMAD7 is a transcriptional target and a negative regulator of TGFβ signalling forming a negative feedback loop. Here the authors show that in cancer cells TGFβ activates the expression of microRNA-182 that suppresses SMAD7 protein, promoting TGFβ-mediated breast tumour invasion and bone metastasis.

## Graphical abstract

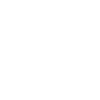

The TGFβ pathway is a key player in embryonic development and cellular homoeostasis in species ranging from flies to mammals. The signalling cascade initiates when the ligands bind to TGFβ type II receptors, which recruit and phosphorylate type I receptors. The type I receptors in turn phosphorylate the receptor-regulated SMADs (R-SMADs), SMAD2 and SMAD3, that form complexes with the common SMAD (Co-SMAD) protein SMAD4 and shuttle into the nucleus. R-SMAD/co-SMAD complexes accumulate in the nucleus and bind to target genes for transcriptional regulation^1^,^2^. The malfunction of TGFβ signalling can result in many pathological changes, among which epithelial-mesenchymal transition (EMT) is a well-studied process that endows cancer cells with increased aggressiveness. EMT refers to the reprogramming of epithelial cells to a mesenchymal-like phenotype, which occurs in many developmental processes such as gastrulation, neurulation and heart morphogenesis^3^,^4^. The process is driven by a set of transcriptional factors, including the zinc finger factors Snail, Slug, ZEB1/2 and FOXC2, and the basic helix-loop-helix factors TWIST and E47. These factors coordinate in an elaborate manner to suppress the expression of the epithelial marker E-cadherin (CDH1) and induce the expression of mesenchymal markers such as N-cadherin (CDH2), Vimentin and Fibronectin. The TGFβ pathway regulates, acting alone or in cooperation with other signalling pathways, these transcription factors, which confers TGFβ a potent inducer of EMT (refs 5, 6). As a physiological phenomenon hijacked by cancer, EMT enhances cancer cell stemness, motility and invasiveness^7^,^8^. In addition to EMT, TGFβ signalling plays roles in other metastasis-related processes, including microenvironment remodelling of target organs for cancer cell metastatic outgrowth. In particular, TGFβ is critical for cancer cell adaptation and colonization of bone. After arriving at bone, cancer cell responds to TGFβ stimulation and promotes osteoclast maturation via secretory factors such as PTHLH. In turn mature osteoclasts cause bone digestion, leading to the release of various growth factors including TGFβ embedded in bone matrix and thus further stimulation of cancer cells, constituting so called osteolytic ‘vicious cycle'^9^,^10^. Thus TGFβ responsiveness is a prerequisite of cancer cells for initiating osteolytic metastasis^9^,^11^,^12^.

Since TGFβ signalling has vital roles in multiple biological processes, the pathway components, including the ligands, the receptors and the SMAD proteins, are tightly regulated by various mechanisms. One such mechanism is mediated by the inhibitory SMAD (I-SMAD) protein SMAD7. SMAD7 can compete with R-SMADs for binding to the type I receptor and prevents their phosphorylation^1^,^13^. It can also recruit SMURF to TGFβ receptors for polyubiquitination and degradative endocytosis^14^. In addition, SMAD7 disrupts the formation of TGFβ-induced SMAD-DNA complex by binding to the SMAD-binding elements (SBE) via its MH2 domain^15^. As *SMAD7* transcription is rapidly induced by TGFβ (refs 13, 16), it represents a negative feedback mechanism for precise control of cellular responses to TGFβ, which is critical during physiological regulation. However, it is unclear whether or how this feedback loop can be disrupted under pathological conditions, especially in cancer cells.

MicroRNAs (miRNAs) are small non-coding RNAs of 19–24 nucleotides in length and exert their regulatory functions by mRNA degradation or translational inhibition. Accumulating evidence demonstrates that miRNAs play critical roles in TGFβ signalling regulation, EMT and cancer metastasis^17^,^18^. Among them, miR-182 has been recently found to promote cancer cell metastasis and also mediate the crosstalk between TGFβ and NFκB pathways^19^. In this study, we report a new role of miR-182 to potentiate TGFβ signalling. miR-182 is induced by TGFβ and targets SMAD7 for translational inhibition. The expression of miR-182 antagonizes the response of SMAD7 to TGFβ and promotes cancer cell EMT, invasion, as well as distant metastasis. Therefore, our study reveals a novel mechanism of cancer cells to disengage the negative feedback chain of TGFβ during metastasis.

## Results

### TGFβ activates SMAD7 transcription but not translation

**Figure 1:**
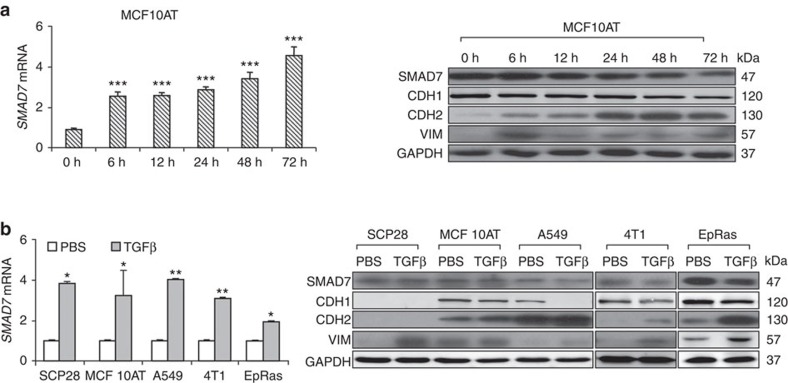
TGFβ stimulation of cancer cells increases *SMAD7* mRNA level but not the protein level. (**a**) *SMAD7* mRNA expression (left), and western blot analyses of SMAD7 and EMT markers (right) after TGFβ stimulation for the indicated time duration in MCF10AT (*n*=3). (**b**) *SMAD7* mRNA expression (left), and western blot analyses of SMAD7 and EMT markers (right) in the indicated cell lines after 72 h of TGFβ treatment (*n*=3). *P<0.05, **P<0.01, ***P<0.001 versus control by student's *t*-test. Error bars are defined as s.d.

TGFβ stimulation usually elicits sustained morphological and behavioural changes of cancer cells, despite the SMAD7-mediated negative feedback of the pathway. To investigate whether the feedback loop actually takes effect in cancer cells, we treated the breast cancer cell line MCF10AT with the TGFβ1 cytokine in a time course of 6–72 h, and then examined the *SMAD7* expression. As expected, the *SMAD7* mRNA was rapidly induced by TGFβ and sustained at high levels during the whole time course. However, when the SMAD7 protein was analysed, it was scantily increased at first but then gradually dropped after 6 h, a pattern in stark contrast to the mRNA changes (Fig. 1a). This phenomenon was also observed in an additional panel of breast and lung cancer cell lines, including SCP28 (ref. 20), MCF10AT, 4T1, EpRas and A549. After TGFβ treatment for 72 h, *SMAD7* mRNA was significantly induced, but the protein was kept unchanged or even slightly down-regulated (Fig. 1b). Meanwhile, TGFβ treatment resulted in obvious and persistent changes of EMT marker proteins in these cells, including the reduction of CDH1, and the increase of CDH2 and Vimentin (Fig. 1a,b, and western blot quantification in Supplementary Fig. 12). Under these circumstances, the SMAD7-mediated negative feedback loop of TGFβ signalling was disrupted in these cancer cell lines, leading to sustained responses of EMT.

### miR-182 is induced by TGFβ and suppresses SMAD7 translation

**Figure 2:**
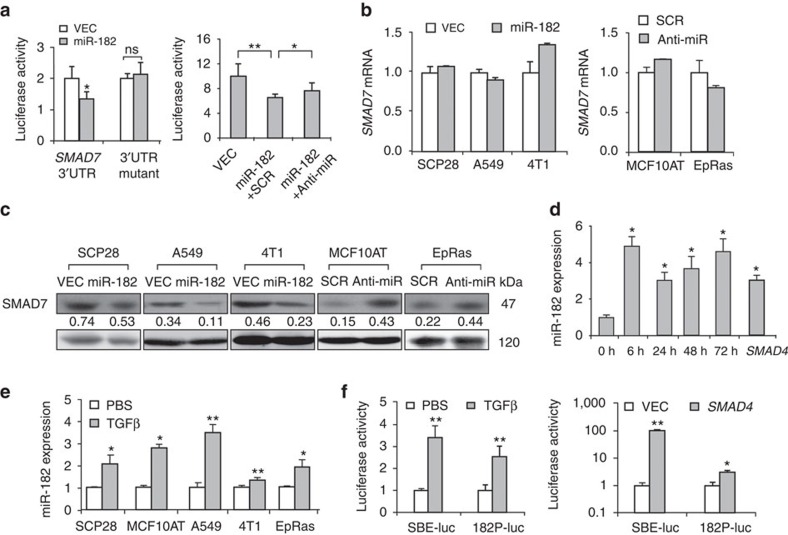
miR-182 directly targets *SMAD7* and is induced by TGFβ. (**a**) Luciferase reporter assays of *SMAD7* wild-type and mutated 3′UTR with miR-182 overexpression and inhibition (*n*=4). (**b**) Endogenous *SMAD7* mRNA levels after miR-182 overexpression and inhibition in the indicated cancer cells (*n*=3). (**c**) SMAD7 protein levels after miR-182 overexpression and inhibition in the indicated cancer cells. Numbers denote western blot quantification of SMAD7 normalized to GAPDH. (**d**) miR-182 expression in SCP28 after TGFβ treatment for the indicated time duration or *SMAD4* transfection (*n*=3). (**e**) miR-182 expression after 48 h of TGFβ stimulation in the indicated cell lines (*n*=3). (**f**) Luciferase reporter assays of *miR-182* promoter in HeLa cells after TGFβ stimulation or *SMAD4* overexpression (*n*=4). The SBE reporter serves as a positive control. Anti-miR, miR-182 oligonucleotide inhibitor; VEC, empty vector control; SCR, scrambled siRNA control. *P<0.05, **P<0.01, ns, not significant by student's *t*-test. Error bars are defined as s.d.

The discrepant expression of *SMAD7* transcript and protein indicated the presence of *SMAD7* post-transcriptional regulation responding to TGFβ. Since miRNA is a widely known mechanism for such regulation, we analysed whether miRNAs were responsible for the suppression of SMAD7 protein. Using the *in silico* miRNA target prediction software TargetScan^21^,^22^, we found several potential miRNA binding sites at the 3′UTR of *SMAD7* (Supplementary Table 1). Among these miRNAs, miR-182 was previously reported to be involved in cancer metastasis and TGFβ signalling by us and others^19^,^23^, and thus represented a promising candidate to interfere TGFβ regulation of *SMAD7*. We cloned *SMAD7* 3′UTR sequence containing the predicted miR-182 binding site for luciferase reporter assays. The luciferase activity of *SMAD7* 3′UTR was significantly reduced on miR-182 transfection in HeLa cells. Moreover, a 3-bp mutation in the seed sequence of the binding site abrogated the reduction of luciferase activity in response to miR-182 (Fig. 2a; Supplementary Fig. 1). In addition, treatment of the cells with an oligonucleotide inhibitor of miR-182 effectively restored the luciferase activity of *SMAD7* 3′UTR (Fig. 2a). Therefore, *SMAD7* 3′UTR is a direct target of miR-182.

To determine whether *SMAD7* was regulated by miR-182 endogenously, we analysed miR-182 expression levels in the breast and lung cancer cell lines (Supplementary Fig. 2A) and assessed the effects of miR-182 overexpression or inhibition in these cells. miR-182 was stably overexpressed in SCP28, 4T1 and A549 (Supplementary Fig. 2B). Although *SMAD7* mRNA was not obviously affected by miR-182 (Fig. 2b), a significant reduction of SMAD7 protein abundance was observed on miR-182 overexpression in these cancer cells (Fig. 2c). In addition, treating MCF10AT and EpRas with an anti-miR-182 oligonucleotide inhibitor^23^ elevated the protein level of SMAD7 but did not affect its mRNA level (Fig. 2b,c). We then used a sponge construct^23^ to inhibit miR-182 in the lung adenocarcinoma cell line NCI-H1299. The inhibition efficacy of the sponge was validated with 3′UTR of a known miR-182 target gene MTSS1 (ref. 23) (Supplementary Fig. 2C). It was found that the sponge also led to in SMAD7 protein elevation (Supplementary Fig. 2D). Therefore, miR-182 suppresses SMAD7 expression at its protein level. In addition, we found that miR-182 neither upregulated the E3 ligase of SMAD7, Arkadia^24^, nor changed the ubiquitination status of SMAD7 protein (Supplementary Fig. 3). Taken together, these data indicate that miR-182 target SMAD7 for translational inhibition.

Previously it was reported that miR-182 expression could be induced by TGFβ in gallbladder cancer and glioma cells^19^,^25^. Altogether with the fact that miR-182 suppresses SMAD7 specifically at the protein level, this could explain the apparent disassociation of *SMAD7* mRNA and protein expression following TGFβ treatment. Therefore, we assessed whether miR-182 could be induced by TGFβ in the above breast and lung cancer cell lines, and found that miR-182 was rapidly induced by TGFβ in SCP28. A nearly 5-fold increment of miR-182 transcript was observed 6 h after TGFβ treatment (Fig. 2d). miR-182 transcription was activated by the transfection of *SMAD4* as well (Fig. 2d). The induction of miR-182 by TGFβ treatment was also confirmed in other breast and lung cancer cell lines (Fig. 2e). *In silico* analysis of the 2.5 kb promoter region of the miR-182 gene revealed several potential SBE sites. Therefore, we constructed the luciferase reporter of this region and found that it was responsive to both TGFβ treatment and *SMAD4* overexpression (Fig. 2f). Thus, miR-182 is both a downstream target of TGFβ and a direct suppressor of SMAD7 in these cancer cell lines.

### miR-182 promotes cancer cell migration and tumour invasion

**Figure 3:**
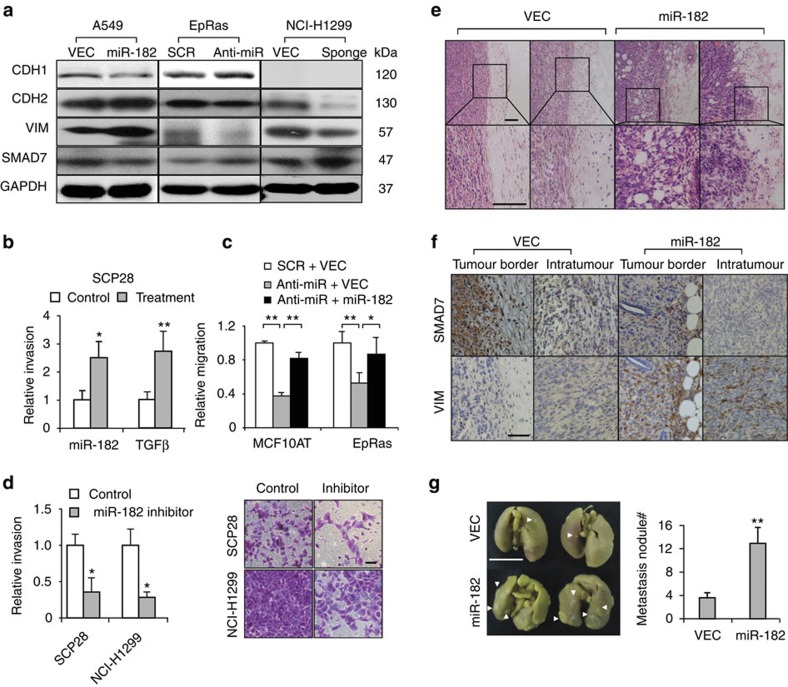
The effects of miR-182 on cancer cell invasiveness and tumour metastasis. (**a**) Protein levels of EMT markers and SMAD7 in cancer cells after miR-182 overexpression or silencing. (**b**) Transwell invasion assays of SCP28 with miR-182 overexpression or TGFβ treatment (*n*=4). (**c**) Migration assays of MCF10AT and EpRas cells with miR-182 silencing and exogenous miR-182 overexpression (*n*=4). (**d**) Transwell invasion assays of cancer cells treated with miR-182 inhibitors. SCP28 was treated with the oligonucleotide inhibitor and NCI-H1299 with the sponge inhibitor (*n*=4). (**e**) Tumour edge H&E staining of 4T1 cells with miR-182 overexpression. (**f**) SMAD7 and Vimentin IHC staining of 4T1 primary tumours. (**g**) Whole-lung images and quantification of metastasis nodules at the lung surface on day 35 after orthotopic injection of 4T1 cells (*n*=10 mice per group). Arrowheads point to metastasis nodules. Scale bars, 1 cm (**g**) and 150 μm (**d**–**f**). *P<0.05, **P<0.01, ns, not significant by student's *t*-test. Error bars are defined as s.d.

The suppression of SMAD7 by miR-182 indicated that miR-182 might play a role in cancer cell EMT, migration and invasion. Therefore, we investigated the effects of miR-182 in these processes. Although we did not observe obvious morphological changes in cancer cells after miR-182 overexpression (Supplementary Fig. 4A), the EMT marker proteins, namely CDH1, CDH2 and Vimentin, were modestly regulated by miR-182. In A549 cells, the overexpression of miR-182 led to a slight reduction of CDH1 and induction of CDH2 and Vimentin. Reciprocally, miR-182 inhibition in EpRas by the oligonucleotide inhibitor resulted in CDH1 up-regulation, as well as CDH2 and Vimentin suppression (Fig. 3a). The sponge inhibitor also led to the suppression of CDH2 and Vimentin in NCI-H1299 (Fig. 3a).

Next, the effects of miR-182 in cancer cell migration and invasion were tested. Forced expression of miR-182 markedly enhanced the transwell invasiveness of SCP28 to a similar extent of TGFβ treatment (Fig. 3b). Meanwhile, miR-182 inhibition with the oligonucleotide or the sponge inhibitor significantly impaired the migration and invasion of MCF10AT, EpRas, SCP28 and NCI-H1299 cells (Fig. 3c,d). In addition, re-introducing miR-182 into the cells by overexpression rescued the impaired cell migration caused by miR-182 inhibition (Fig. 3c). Taken together, these data demonstrate that though miR-182 has a weaker effect on EMT regulation, it mimics TGFβ signalling to promote cancer cell migration and invasion.

To further assess the function of miR-182 in tumour invasion and metastasis *in vivo*, we used the orthotopic transplantation model of 4T1 breast cancer cells. 4T1 cells with stable miR-182 overexpression and the control cells were implanted into the mammary fat pads of BALB/c mice. No difference in the rate of primary tumour growth was detected (Supplementary Fig. 4B). However, when the tumours were histologically examined, we observed markedly enhanced invasiveness of the miR-182-expressing tumours. These tumours had widely-spread invasive fronts characterized by infiltrated tumour–stroma interface and the presence of tumour cells intermingled in the stromal side. In contrast, the control tumours were well contained with intact tumour–stroma interface (Fig. 3e). The augmented invasiveness was accompanied by attenuated SMAD7 and enhanced Vimentin both at the edge and the interior areas of the tumours, as revealed by immunohistochemistry (IHC) analyses (Fig. 3f). In addition, when the mice were euthanized and their lungs were examined at the 5th week post tumour transplantation, significantly more metastases were observed in the overexpression group. The number of metastasis nodules on the pulmonary surface was over 3-fold higher than the control (Fig. 3g). These findings demonstrated a functional role of miR-182 to promote tumour invasion and spreading *in vivo*.

### miR-182 is required for TGFβ-induced EMT and invasion

**Figure 4:**
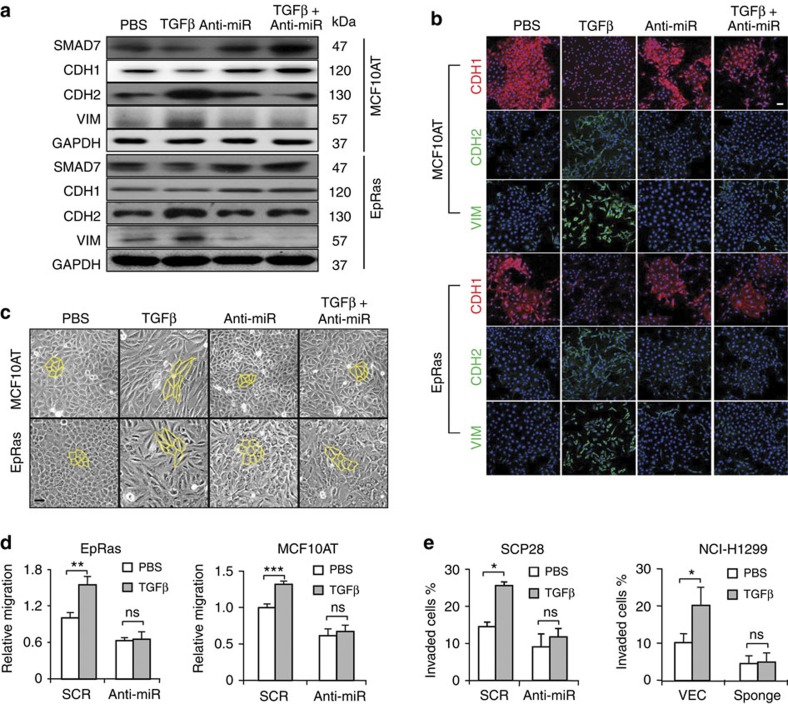
miR-182 silencing impairs TGFβ-induced EMT and invasion. (**a**) Protein expression of EMT markers and SMAD7 in EpRas and MCF10AT cells after TGFβ stimulation and miR-182 inhibition. (**b**) Immunofluorescence analyses of EMT makers in EpRas and MCF10AT. (**c**) Morphological changes of EpRas and MCF10AT cells. (**d**) Migration of EpRas and MCF10AT cells (*n*=4). (**e**) Transwell invasion of SCP28 and NCI-H1299 cells (*n*=4). Scale bars, 50 μm. *P<0.05, **P<0.01, ***P<0.001 by student's *t*-test. Error bars are defined as s.d.

Since miR-182 suppressed TGFβ-induced upregulation of SMAD7, we reasoned that this miRNA could be critical for cancer cell responses to TGFβ. Therefore, we treated MCF10AT and EpRas with the anti-miR-182 oligonucleotide together with TGFβ, and analysed the cellular responses in EMT. Western blot and immunofluorescence analyses showed that miR-182 inhibition resulted in upregulation of SMAD7 protein, and suppressed the changes of EMT markers in response to TGFβ in the cancer cells (Fig. 4a,b). Without miR-182 inhibition, TGFβ treatment markedly reduced the epithelial marker CDH1 that was localized to the cell membrane, while the mesenchymal markers CDH2 and Vimentin were significantly elevated (Fig. 4b). However, these TGFβ-induced molecular events were completely abolished when the cells were simultaneously treated by the miR-182 inhibitor (Fig. 4b). Such changes in the expression of EMT markers, as well as EMT-related transcription factors such as TWIST1 and ZEB1, were also observed by western blot and mRNA qPCR analyses in these cells (Fig. 4a, Supplementary Figs 5 and 6). Notably, miR-182 inhibition also quenched the morphological shift of cancer cells induced by TGFβ. TGFβ treatment led the cancer cells transform from the cobble stone-like appearance to the elongated, spindle-like mesenchymal shape. In contrast, no manifest changes were observed in cell morphology after the treatment of TGFβ along with the inhibitor (Fig. 4c).

In addition to the observations of molecular and morphological changes, miR-182 silencing effectively suppressed cell migration and invasion caused by TGFβ in EpRas, MCF10AT, SCP28 and NCI-H1299. In the presence of miR-182 inhibitors, TGFβ failed to enhance the motility or invasiveness of cancer cells (Fig. 4d,e). Thus, these data suggest that miR-182 is critical for TGFβ responses of cancer cells in EMT and invasion.

As a comparison, we also analysed the role of miR-182 in TGFβ responses of non-malignant mammary epithelial cells MCF10A and HMLE. Unlike in cancer cells, TGFβ treatment of these normal cells failed to induce the expression of miR-182, and thus an obvious increase of SMAD7 protein was observed following TGFβ stimulation (Supplementary Fig. 7A,B). Concordantly, miR-182 did not obviously alter the TGFβ responsiveness of the SBE reporter, or TGFβ-induced cell apoptosis in MCF10A (Supplementary Fig. 7C,D). Therefore, the positive feedback loop of TGFβ-miR-182 seems to be specific to cancer cells.

### miR-182 promotes EMT and invasion by suppressing SMAD7

**Figure 5:**
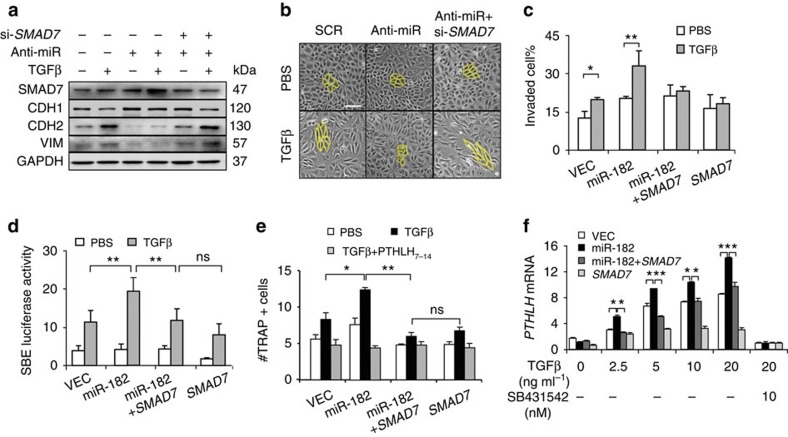
SMAD7 rescued the effect of miR-182 in TGFβ-induced EMT and osteoclastogenesis. (**a**) Protein levels of EMT markers and SMAD7 in EpRas with TGFβ stimulation, miR-182 inhibition and *SMAD7* silencing. (**b**) EMT morphological changes of EpRas with TGFβ stimulation, miR-182 inhibition and *SMAD7* silencing. (**c**) Transwell invasion of SCP28 with TGFβ stimulation, miR-182 and *SMAD7* overexpression (*n*=4). (**d**) SBE luciferase reporter assays of HeLa cells with TGFβ stimulation, miR-182 and *SMAD7* overexpression (*n*=4). (**e**) Osteoclastogenesis of primary bone marrow in conditioned medium of SCP28 with miR-182 and *SMAD7* overexpression. Tumour cells were treated with TGFβ, followed by conditioned medium harvest and primary bone marrow culture. The peptide antagonist PTHLH_7-34_ (0.5 μM) was added into the bone marrow culture (*n*=4). (**f**) *PTHLH* expression in SCP28 treated with TGFβ at different concentration, and the TGFβ inhibitor SB431542 (*n*=3). Scale bars, 100 μm. *P<0.05, **P<0.01 by student's *t*-test. Error bars are defined as s.d.

To corroborate that SMAD7 suppression mediates the role of miR-182 in cancer cell TGFβ responses, we treated EpRas cells with a *SMAD7* siRNA inhibitor (Supplementary Fig. 8A) in addition to TGFβ stimulation and miR-182 inhibition. While miR-182 suppression led to SMAD7 induction on TGFβ treatment and abolished TGFβ-induced EMT phenotypes, simultaneous treatment with the *SMAD7* inhibitor restored cancer cell responses to TGFβ. The SMAD7 protein level was effectively suppressed, along with the manifest changes of EMT markers and cellular morphology after TGFβ stimulation, despite the inhibition of miR-182 (Fig. 5a,b). The assays were repeated in MCF10AT cells as well, and the same changes in EMT markers were observed (Supplementary Fig. 8B), suggesting that SMAD7 induction mediates the effects of miR-182 inhibition in cancer cell EMT.

Reciprocally, we stably overexpressed both *SMAD7* and miR-182 in SCP28 (Supplementary Fig. 8C), followed by TGFβ treatment. As expected, *SMAD7* overexpression abolished the effects of miR-182 in cancer cell invasion. With forced expression of *SMAD7*, miR-182 was no longer able to boost the cancer cell invasion after TGFβ stimulation (Fig. 5c). Similarly, treating the cells with the TGFβ receptor inhibitor SB431542 also abolished the effect of miR-182 on cell invasion (Supplementary Fig. 8D). The same phenomenon was observed in 4T1 cells when miR-182 and *SMAD7* were both overexpressed, following assessments of cell migration and invasion (Supplementary Fig. 8E). We also analysed the TGFβ response of SCP28 cells with SBE luciferase reporter assays. TGFβ treatment induced the SBE reporter activity, and miR-182 overexpression further enhanced the response. In contrast, *SMAD7* diminished the enhancing effect of miR-182 on SBE activity, although the reporter was still partially responsive to TGFβ stimulation. Importantly, in the presence of *SMAD7* overexpression, miR-182 failed to enhance the SBE response to TGFβ (Fig. 5d). These data showed that miR-182 promotes cancer cell responses to TGFβ by suppressing SMAD7.

### miR-182 promotes osteoclastogenesis and bone metastasis

In addition to induction of EMT and cancer cell invasion, TGFβ signalling also underlies the process of skeleton microenvironment remodelling and osteoclast differentiation induced by cancer cells for metastasis to bone, a physiological reservoir of TGFβ. Therefore, we further analysed the role of miR-182 in TGFβ signalling during bone metastasis. We first assessed the ability of cancer cells to induce the differentiation of pre-osteoclasts in murine bone marrow. Primary marrow was cultured in the conditioned medium from SCP28 cells with miR-182 and/or *SMAD7* overexpression and osteoclast maturation was assessed by tartrate-resistant acid phosphatase (TRAP) staining. As expected, TGFβ treatment of SCP28 before conditioned medium harvest promoted osteoclast maturation when bone marrow was cultured in the conditioned medium (Fig. 5e; Supplementary Fig. 9A). In contrast, adding TGFβ directly into the bone marrow culture produced no difference of osteoclastogenesis (Supplementary Fig. 9B). Notably, miR-182 overexpression was able to augment cancer cell response to TGFβ for osteoclast induction. However, *SMAD7* expression completely blocked the effect of miR-182 in osteoclast maturation after TGFβ treatment (Fig. 5e). Previously we and others have showed that cancer cells, in response to TGFβ, express and secret PTHLH, which stimulates *RANKL* expression of osteoblasts and induces osteoclast maturation^9^,^26^,^27^,^28^. Concordantly, miR-182 overexpression in SCP28 enhanced *PTHLH* expression and secretion of SCP28 following TGFβ treatment, as well as *Rankl* expression of the pre-osteoblast C2C12 cells when cultured in SCP28 conditioned medium, while *SMAD7* or the TGFβ inhibitor SB431542 abrogated such effects of miR-182 (Fig. 5f, Supplementary Fig. 10A,B). In addition, other TGFβ target genes, such as *IL11* (ref. 10), were enhanced by miR-182 and suppressed by SMAD7 in SCP28 as well. In contrast, *CSF1*, which was also involved in osteoclastogenesis^10^ but was not responsive to TGFβ, was not regulated by miR-182 either (Supplementary Fig. 10C). These observations corroborated that miR-182 enhances TGFβ signalling of cancer cells. We also found that the peptide inhibitor of PTHLH, PTHLH_7–14_ (ref. 9), could completely block the osteoclastogenesis effect of miR-182 (Fig. 5e), indicating that PTHLH is the main downstream mediator of miR-182 to regulate TGFβ-induced osteoclast maturation.

**Figure 6:**
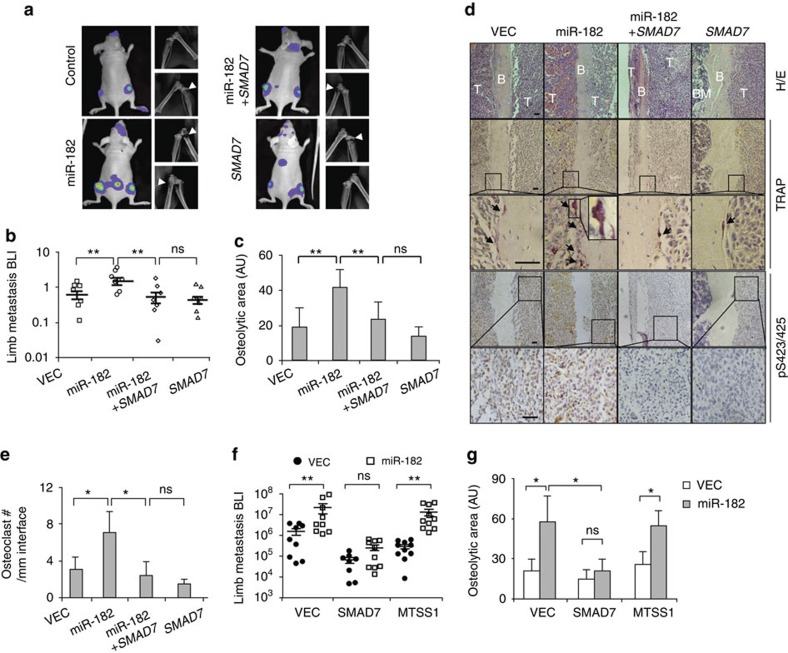
The role of miR-182 in bone metastasis. (**a**) Bioluminescent imaging (BLI) and X-ray of bone metastases by SCP28 cells. Arrowheads denote areas of overt osteolysis. (**b**) BLI quantitation of limb metastasis by SCP28 4 weeks after intracardiac injection (*n*=7). (**c**) Quantitation of osteolytic areas caused by SCP28 (*n*=7). (**d**) Representative H&E images, osteoclast TRAP staining, and IHC analyses of SMAD3 phosphorylation of bone metastases by SCP28. Arrows point to TRAP+ osteoclasts along the tumour–bone interface. Scale bars, 50 μm. (**e**) Osteoclast numbers along the tumour–bone interface (*n*=7). (**f**) BLI quantitation of limb metastasis by SCP28 4 weeks after intratibial injection (*n*=10). (**g**) Quantitation of osteolytic area sizes caused by SCP28 cells after intratibial injection (*n*=10). AU, arbitrary unit. *P<0.05, **P<0.01 by student's *t*-test. Error bars are defined as s.d.

Then, we analysed the consequences of miR-182 and *SMAD7* overexpression in breast cancer bone metastasis *in vivo*. The miR-182-overexpressing or control SCP28 cells were intracardially injected into the circulation of nude mice followed by bioluminescent imaging of bone metastasis. First we confirmed that miR-182 was still overexpressed in the metastasis tumours caused by miR-182-overexpressing cancer cells (Supplementary Fig. 11A). miR-182 overexpression significantly accelerated the outgrowth of cancer cells in bone, and caused more severe bone damages (Fig. 6a–c). Interestingly, *SMAD7* reversed the phenotypes and dampened bone metastasis. More importantly, with forced expression of *SMAD7*, miR-182 caused no obvious difference in the formation of bone metastases (Fig. 6a–c). Concordant to the difference in bone damages, there were significantly more osteoclasts along the tumour–bone interface when the tumour cells overexpressed miR-182, and this increment was suppressed by *SMAD7* overexpression (Fig. 6d,e). Additionally, miR-182 and SMAD7 led to opposite changes in the level of SMAD3 phosphorylation and PTHLH expression in bone metastases (Fig. 6d; Supplementary Fig. 11B).

To further validate the role of miR-182 in bone metastasis of cancer cells, SCP28 cells with concurrent overexpression of miR-182 and its target genes were injected intratibially into the bone marrow of mice, followed by analyses of tumour growth and bone damage. Again, we found that miR-182 accelerated the growth of cancer cells in bone and osteolytic damage of the skeleton. *SMAD7* overexpression completely blocked such effects of miR-182. In contrast, *MTSS1*, the other miR-182 target gene that also mediates the pro-invasive function of miR-182 (ref. 23), had no effects on SCP28 outgrowth in bone (Fig. 6f,g, Supplementary Fig. 11C). Taken together, these data suggest that miR-182 upregulates cancer cell response to microenvironmental TGFβ and bone metastasis by suppressing SMAD7.

### miR-182 inversely correlates with SMAD7 in clinical samples

**Figure 7:**
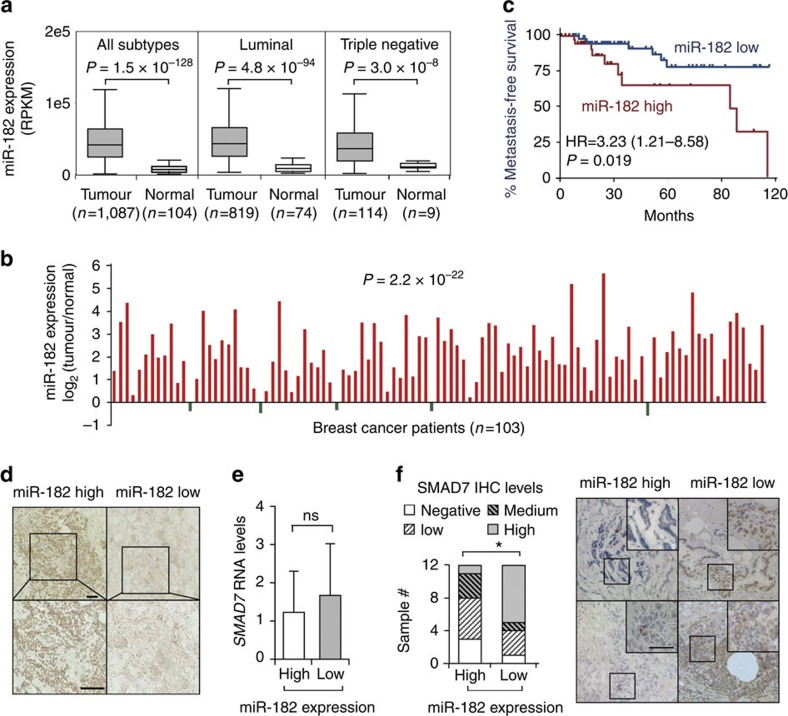
miR-182 was upregulated and negatively correlated with SMAD7 protein in clinical breast tumours. (**a**) Comparison of miR-182 expression in TCGA breast invasive carcinoma and normal tissues. (**b**) Pair-wise comparison of TCGA breast invasive carcinoma and normal tissues of the same patients. Log-ratios of miR-182 expression were shown. (**c**) Metastasis-free survival of the TCGA triple negative breast cancer patients with high or low levels of miR-182 expression. (**d**) miR-182 *in situ* hybridization of the Qilu cohort samples with different miR-182 levels. (**e**) qPCR analyses of *SMAD7* mRNA in the Qilu cohort of human breast tumour samples (*n*=24) with different miR-182 levels. (**f**) SMAD7 protein levels in Qilu samples with different miR-182 levels. Representative IHC images were shown on the right. Scale bars, 50 μm. *P<0.05, ns, not significant by student's *t*-test. Error bars are defined as s.d.

We analysed the expression of miR-182 in clinical breast tumour samples of The Cancer Genome Atlas (TCGA) database and found that miR-182 expression was significantly higher in breast tumours than in normal tissues. The difference was still significant when the analyses were performed for individual breast cancer molecular subtypes (Fig. 7a). A direct comparison of paired tumour and normal tissues of the same patients also revealed upregulation of miR-182 in most of the cases (Fig. 7b). The correlation of miR-182 expression with clinical metastasis was also analysed for the TCGA cohort, and it was found that miR-182 elevation was linked to accelerated metastasis in triple negative breast cancer, a subtype characterized with poor prognosis (Fig. 7c). To further investigate the clinical relevance of SMAD7 suppression by miR-182, we examined a cohort (*n*=24) of breast cancer clinical samples collected from Qilu Hospital by q-PCR of miR-182 and IHC staining of SMAD7. qPCR analysis of miR-182 expression in these tumour samples were first validated by *in situ* hybridization analysis with a miR-182 probe (Fig. 7d). Although we did not observe obvious differences of *SMAD7* mRNA levels in the tumours expressing high or low levels of miR-182 (Fig. 7e), the SMAD7 protein level was significantly lower in the samples with abundant miR-182. Only 1 out of the 12 samples with high levels of miR-182 showed strong staining of SMAD7, as compared with 7 of the 12 samples with low levels of miR-182 (*P*=0.027, Fig. 7f), corroborating an inverse correlation between SMAD7 protein and miR-182. The linkage of miR-182 expression with the SMAD7 protein, but not with the *SMAD7* mRNA, was concordant to our observation that miR-182 targeted SMAD7 to suppress its protein level (Fig. 2b,c). Collectively, our data support the conclusion that miR-182 targets SMAD7 to enhance TGFβ signalling in cancer cell lines and human tumour samples.

## Discussion

Since the identification of SMAD7 as a negative regulator of TGFβ (ref. 29), it has been assumed to serve as a brake of TGFβ signal transduction. Indeed, the *SMAD7* mRNA can be rapidly induced up to 4–10 folds by TGFβ treatment in numerous types of cells^30^,^31^; meanwhile, upregulation of SMAD7 to the same magnitude in mucosal mononuclear cells of inflammatory bowel disease patients as compared with the normal controls was sufficient to desensitize the cells to TGFβ (ref. 32). Forced expression of *SMAD7* in cancer cells also blocked the cellular responses to TGFβ (ref. 33). These observations support a role of TGFβ-induced SMAD7 to limit the propagation of TGFβ signalling. However, it is also well known that TGFβ treatment of cancer cells usually instigates a seemingly unchecked responses, including altered gene expression, EMT, invasiveness and metastasis microenvironment remodelling^1^,^34^. These manifest and persistent changes raise the question regarding whether and how cancer cells can avert the turnoff signal from I-SMADs. Although it has been well established that *SMAD7* mRNA can be activated by TGFβ (refs 16, 29, 30, 35, 36, 37), only very few studies reported the elevation of SMAD7 protein following TGFβ stimulation in cancer cells^38^. In addition, most of the functional studies of *SMAD7* that validated its TGFβ-inhibitory role in cancer have been carried out with ectopic overexpression of the gene^33^,^39^,^40^,^41^,^42^, thus not necessarily reflecting what actually occurs in cancer cells after TGFβ treatment. In this study we observed an unexpected phenomenon in a panel of breast and lung cancer cell lines that in spite of *SMAD7* mRNA induction in immediate response to TGFβ, the protein abundance of SMAD7 was not elevated, or even slightly reduced following the stimulation (Fig. 1c), indicating the inefficiency of SMAD7-mediated feedback to restrain TGFβ signalling in these cancer cells and in the studied context. We further found that these cancer cells achieved this via TGFβ-induced expression of miR-182, which suppressed SMAD7 and thus endorsed the extended activation of TGFβ signalling leading to EMT and metastasis. Concordantly, miR-182 silencing with oligonucleotide or sponge inhibitors reactivated the self-limiting signal of the TGFβ pathway and impaired cancer cell responses in morphological transformation and microenvironment remodelling. Thus our study reveals a route of cancer cells to circumvent the abnegation of TGFβ signalling, and provides an explanation of sustained responses of cancer cells to TGFβ stimulation. A remaining question is whether this route is specific to the studied cell lines or other cancer cells could also adopt the same scheme to enhance TGFβ responses.

Balance of positive and negative inputs is crucial to maintain the proper functions of signal transduction. However, cancer cells are often characterized by the loss of control of signal pathways that govern cell proliferation and malignant progression^43^. The miR-182-mediated disruption of TGFβ negative feedback provides a good example of how central pathways can be disregulated in malignant cells. Rationally, another way to break the loop holding back TGFβ signalling would be genetic mutations of SMAD7, which have been reported in cancers^44^,^45^. However, missense and nonsense SMAD7 mutations have been mainly observed in colorectal cancer and are not linked to increased tumour metastasis^45^,^46^,^47^. Thus our findings reveal a novel mechanism for cancer cells to enhance TGFβ signalling and promote metastasis. It remains to be further studied what happens in normal cells, although our preliminary results showed that in non-cancerous mammary epithelial cells MCF10A and HMLE TGFβ treatment did not result in miR-182 elevation and thus the SMAD7 protein was induced (Supplementary Fig. 7). Therefore, the positive feedback loop of TGFβ signalling and miR-182 expression seems to be disconnected in normal epithelial cells, although the mechanism of insensitivity of miR-182 expression to TGFβ treatment in these cells is unclear.

As SMAD7 is a critical component in the TGFβ pathway, understanding how it is regulated is crucial to elucidate the role and mechanism of TGFβ signalling. While *SMAD7* is known to be transcriptionally regulated by TGFβ and NFκB pathways^29^,^48^,^49^, recent studies have reported that post-transcriptional regulation, such as protein acetylation and ubiquitination, plays key roles to control its protein level^50^,^51^. Accumulating evidence has also shown the involvement of miRNAs, including miR-21, miR-106-25 and miR-216a/217, in the regulation of SMAD7 (refs 52, 53, 54). In this study, we showed that miR-182 also targets SMAD7 to elevate cancer cell responses to TGFβ during metastasis. Notably, miR-182 selectively inhibits the protein level of SMAD7, but not its transcript level, which was confirmed by our expression analysis of clinical samples. In addition, the lack of correlation between I-SMAD mRNA and protein levels was recently noticed in pancreatic cancer^55^. Although multiple IHC analyses of SMAD7 have firmly established the correlation of SMAD7 protein expression with better prognosis of cancer patients^56^,^57^,^58^, the *SMAD7* mRNA levels did not show such clinical relevance^59^,^60^. Collectively, these studies underscore the importance of post-transcriptional regulation of *SMAD7* to coordinate TGFβ signalling in developmental and pathological conditions.

miR-182 is a pleiotropic miRNA that regulates circadian rhythm, immune system and DNA repair^61^,^62^,^63^,^64^. Its role in cancer has also been reported^19^,^23^,^65^,^66^,^67^,^68^,^69^. Although the majority of these studies demonstrated its pro-metastatic role in melanoma, glioma, ovarian cancer and breast cancer^19^,^23^,^65^,^66^,^67^, two recent studies indicated an opposite function of miR-182 in cancer cell migration and invasion^68^,^69^, suggesting the contextual dependence of its function in cancer metastasis. In this study we, for the first time, report a role of miR-182 in targeting SMAD7 to enhance TGFβ signalling and metastasis. The analyses were performed in multiple cell lines of breast and lung cancers, thus corroborating the findings of earlier reports^19^,^23^,^65^,^66^,^67^. Noticeably, our data showed that although miR-182 was required for TGFβ-induced EMT, its overexpression only led to modest changes in the expression of EMT markers (Fig. 3a), and the regulation in cancer cell morphology was not obvious (Supplementary Fig. 4A). The role of miR-182 in osteoclast induction was also observed only with the supplement of TGFβ *in vitro*, or in bone where TGFβ is largely available. Therefore, miR-182 was a much less potent inducer of EMT than TGFβ itself, suggesting that SMAD7 suppression alone was not sufficient for fully activation of TGFβ signalling. In addition, it is also noticed that *SMAD7* overexpression failed to completely block cancer cell TGFβ responses as revealed by SBE activity, osteoclast differentiation and PTHLH induction (Fig. 5d–f), implying a SMAD7-independent mechanism for the oncogenic role of miR-182 and the TGFβ response of cancer cells. For example, the promotion of cell invasiveness by miR-182 might result from the synergistic action of SMAD7 and other downstream targets, such as MTSS1 (ref. 23).

miR-182 not only regulates TGFβ signalling by targeting SMAD7, but also is responsive to TGFβ signalling, thus constituting a self-enhancing circuit of the pathway. The TGFβ negative feedback loop mediated by I-SMADs has been well studied, and now our study show that this loop is antagonized by the miRNA-mediated positive feedback signal, which expands the spectrum of TGFβ regulatory network and further reveals the complexity of this pathway in cancer progression. In addition, our data demonstrated the aberrant up-regulation of miR-182 and its negative correlation with SMAD7 protein in tumours samples. Previously we also showed that miR-182 expression was linked to elevated risk of metastasis in breast cancer patients^23^. Overall, our results demonstrate that miR-182 is a critical component of TGFβ signalling. The uncovering of this TGFβ-miR-182 circuit will extend our comprehension of TGFβ network complexity and argue for miR-182 as a new option to target TGFβ signalling for cancer intervention.

## Methods

### Plasmid and cell lines

Overexpression of miR-182 and MTSS1 was performed in the pMSCV retroviral plasmid^23^. The miR-182 sponge plasmid was constructed by inserting eight tandemly arrayed miR-182-binding sites (50-AGTGTGAGTTCTAGGGTTTGCCAAA-30) into the 3′UTR of dsRed^23^. For 3′UTR reporter assays, a 0.8 kb fragment of *SMAD7* 3′UTR was cloned into pMIR-REPORT (Ambion) with SpeI and SacI digestion. The miR-182 seed sequence was further mutated to 5′-ATGGGTAAT-3′. The 2.1 kb fragment of MTSS1 3′UTR was cloned into pMIR-Report^23^. For promoter reporter assays, a 2.5 kb fragment upstream of the miR-182 gene was cloned into pGL3basic (Ambion) with NheI and XhoI digestion. The cDNA vector of human *SMAD7* was provided by Dr Heldin^29^ and was subcloned to the pMSCV-hygro vector. All constructs were confirmed by sequencing. The plasmids were transiently transfected to target cells with lipofectamin 2000 (Invitrogen, cat.11668). To generate stable lines of overexpression or sponge knockdown, the plasmids were packaged into retrovirus with the amphotropic Phoenix packaging cell line and infected into target cells, followed by puromycin/hygromycin selection of the infected cells. MCF10A and MCF10AT and 4T1 cell lines were obtained from Dr Miller^70^. EpRas was obtained from Dr Weinberg^8^, SCP28 from Dr Massague^20^. NCI-H1299 (TCHu160) and A549 (TCHu150) were purchased from the Cell Bank of Type Culture Collection of Chinese Academy of Sciences. All the cell lines were confirmed as mycoplasma free by mycoplasma PCR tests.

### Reagents

The mouse monoclonal SMAD7 antibody (R&D, cat. MAB2029), mouse VIM antibody (BD Biosciences, cat. 550513) and mouse VIM antibody (Sigma, cat. V2258) were used for western blot and immunohistochemistry analyses. The mouse CDH2 antibody (BD Biosciences, cat. 610920), rabbit CDH1 antibody (Cell Signaling Technology, cat. 3195S), rabbit Arkadia antibody (Proteintech, cat. 14698-1-AP), mouse Ub (P4D1) antibody (Santa Cruz, SC-8017), rabbit MTSS1 antibody (CST, cat. 4385s), goat anti-rabbit IgG (Merck) and goat anti-mouse IgG (Merck) antibodies were used for western blot analyses. The mouse CDH2 antibody (BD Biosciences, cat. 610920), rabbit CDH1 antibody (Cell Signaling Technology), mouse VIM antibody (BD Biosciences, cat. 550513) and the Tyramide Signal Amplification kit (Shanghai EU-BIO, cat. EUT0168) were used for immunofluorescence analyses. Antibody dilutions were 1:500 for primary antibodies and 1:2,000 for secondary antibodies in immunohistochemistry analyses, 1:1,000 for primary antibodies and 1:5,000 for secondary antibodies in western blotting, and 1:200 for primary antibodies in immunofluorescence analyses. The biotinylated horse anti-mouse IgG antibody (Vector Labs) was used for immunohistochemistry analyses. The anti-miR-182 oligonucleotide (5′-AGUGUGAGUUCUACCAUUGCCAAA-3′) and scrambled control oligonucleotide (5′-CAGUACUUUUGUGUAGUACAA-3′) were previously reported^23^ and purchased from GenePharma (Shanghai, China). SMAD7 siRNA (5′-AGGUCACCACCAUCCCCACTT-3′) and the scrambled control (5′-UUCUCCGAACGUGUCACGUTT-3′) were purchased from GenePharma. The DIG-labeled miRCURY LAN detection probes (Exiqon, 38489-01) and anti-Digoxigenin-POD, Fab fragments (Roche, cat. 11207733910) were used for *in situ* hybridization of miR182. MG-132 (Beyotime, s174) was used to inhibit the proteasome activity. The concentration for recombinant human TGFβ1 (R&D) stimulation was 20 ng ml^−1^. The duration for TGFβ and MG-132 treatments was 48 h unless specified.

### miRNA detection

Total RNA was extracted using TRIzol (Invitrogen). Mature miRNAs were reverse-transcribed and quantitated with the TaqMan microRNA Assays (Applied Biosystems). The data was normalized to U6 expression.

### Luciferase dual-reporter assays

HeLa cells were co-transfected with the miR-182-expressing plasmid or control vector, the indicated firefly luciferase reporter plasmids and a renilla luciferase plasmid with a ratio of 2:2:1. Lysates were collected 72 h after transfection. Firefly and renilla luciferase activities were measured with a Dual-Luciferase Reporter System (Promega).

### Invasion assays

Cancer cells of 5 × 10^4^–10^5^ in serum-free medium were seeded into the upper chamber of transwell insert membranes of an 8 μm pore size (Corning) coated with Matrigel (BD Biosciences) in a 24-well plate. Approximately 10% fetal bovine serum (FBS) was used in the bottom chamber as the attractant. The cells in the upper chamber were removed 16–96 h later using a cotton swab. The invaded cells at the lower chamber were stained with crystal violet and counted. For TGFβ1 treatment, the cytokine (20 ng ml^−1^) was added into the upper chamber before the transwell invasion analysis.

### Immunofluorescence analysis

Cancer cells of ∼30% confluence were fixed with PBS containing 3.7% paraformaldehyde without methanol. The cells were washed and permeabilized with 0.2% Triton X-100 and blocked with 3% FBS for 30 min at room temperature. Then immunofluorescence staining was performed with the Tyramide Signal Amplification kit following manufacturer's protocol. Briefly, samples were incubated with the primary antibody overnight at 4 °C, and then were treated with 4% H_2_O_2_ for 15 min, followed by incubation with POD-conjugated anti-mouse or anti-rabbit IgG for 1 h at room temperature. Following washing, samples were incubated with Rhodamine-conjugated tyramide for 15 min. 4,6-diamidino-2-phenylindole (DAPI) was used to counter-stain the nuclei. Pictures were visualized by three-dimensional confocal microscopy (LSM-510META, Carl Zeiss) and acquisition parameters were kept constant for all of the experiments.

### Flow cytometry analysis

Cell apoptosis was determined by flow cytometry analysis. Cells were cultured in the absence or presence of 20 ng ml^−1^ TGFβ1 for 24 h. Cells were collected, washed with cold PBS, fixed in cold 70% Ethanol, treated with DNase-free RNase (Sangon, RB473. 100 μg ml^−1^) and stained with 50 μg ml^−1^ Propidium iodide (Sangon, cat. P1112) and Annexin V-APC/7-AAD kit (KeyGEN, cat.KGA-1025). The cells were analysed using a Gallios flow cytometer (Beckman Coulter) to quantify the proportion of cells in apoptosis status.

### Animal experiments

To study primary tumour growth and lung metastasis, 10^5^ 4T1 cancer cells were resuspended in 1:1 PBS and matrigel mixture and injected into the fat pads of fourth mammary glands of female BALB/c mice. Tumour growth was monitored twice a week by size measurement. Both maximum (L) and minimum (W) diameters of the tumours were measured using a slide caliper, and the tumour volume was calculated as πLW^2^/6. The tumours were surgically removed when reaching the size of 1 cm, and fixed by 4% paraformaldehyde for histological staining to analyse the local invasion. For lung metastasis analysis, all the mice were euthanized after 5 weeks post transplantation, and the lungs were fixed with Bouin's fixative. Then the metastasis nodules on the lung surfaces were counted. Ten mice per group were used for the above analyses.

To study bone metastasis, 10^5^ SCP28 cells were intracardially or intratibially injected to female nude mice. Bioluminescent imaging was acquired with a NightWOL II LB 983 Imaging System (Berthold). Bone damages were detected by X-ray radiography with a Faxitron instrument (Faxitron Bioptics) as previously described _ENREF_9. Osteolytic areas were identified on radiographs as demarcated radiolucent lesions in bone and quantified using ImageJ. Forelimb and hindlimb long bone of mice were excised, fixed in 10% neutral-buffered formalin after 24 h, decalcified (10% EDTA, 2 weeks), dehydrated through a graded alcohol series, embedded in paraffin and stained with H&E. All animal experiments were performed according to the guidelines for the care and use of laboratory animals and were approved by the Institutional Biomedical Research Ethics Committee of Shanghai Institutes for Biological Sciences.

### Osteoclastogenesis assays

Primary bone marrow osteoclastogenesis analysis was performed with bone marrow from 4-to-6-week-old BALB/c mice. Bone marrow cells were harvested and plated in basal culture medium overnight. The next day, non-adherent cells were added at 5 × 10^5^ per well to 24-well plates that were previously seeded with either control or indicated tumour cells supplemented with 25 ng ml^−1^ RANKL(PeproTech) and 25 ng ml^−1^ M-CSF(PeproTech). Medium was changed every 3 days. Cancer cells were treated with 10 ng ml^−1^ TGFβ for 72 h, and the resultant conditioned medium were mixed with α-MEM at 1:9 ratio for bone marrow culturing. TRAP staining was performed with a TRAP kit (387A, Sigma-Aldrich) on day 10–12. Osteoclasts were defined as TRAP-positive cells containing more than three nuclei.

### Clinical samples and analyses

Fresh-frozen and paraffin-embedded tumour specimens were obtained Qilu Hospital of Shandong University with informed consent from all subjects and approval from the Institutional Research Ethics Committee. RNA was extracted from the fresh-frozen tumours and the quality was monitored by O.D. reading. miR-182 and SMAD7 RNA levels were measured by qPCR.

Paraffin-embedded tumour specimens were cut at a thickness of 5 μm. Then the sections were deparaffinized by Xylene and dehydrated through a graded alcohol series. The sections were incubated for 20 min in 3% hydrogen peroxide blocking solution and probed with the SMAD7 antibody (1:125) or the VIM antibody (1:200). Following extensive washings, sections were incubated for 1 h in the secondary antibody (1:200). Following washings, the Avidin Biotin Complex (Vector Labs) was then applied to the sections, followed by extensive washing steps. The DAB Peroxidase Substrate Kit (Vector Labs) was then added to the sections, and incubated for 2–3 min. Sections were then counterstained in hematoxylin (Sigma) and dehydrated in ascending grades of methanol before clearing in xylene and mounting under a cover slip. Finally each sample was scored as negative (0), low (1), medium (2) or high (3) according to staining intensities.

For miR-182 *in situ* hybridization, OCT-embedded fresh-frozen specimens were cut at a thickness of 10 μm and fixed in 10% neutral-buffered formalin at room temperature overnight. The sections were treated with Proteinase K (15 μg ml^−1^) at 37 °C for 15 min, and incubated in the hybridization mix containing the miR-182 probe at 56 °C for 1 h. Following stringent washing in SSC, the slides were incubated with 3% FBS as blocking solution for 30 min at room temperature. The sections were treated with 3% H_2_O_2_ for 15 min in order to quench the endogenous peroxidase. Then the sections were applied with anti-DIG-POD (1:1,000). Following washings the DAB peroxidase substrate was applied to the sections for 5 min. Sections were then counter-stained in hematoxylin and mounted under a cover slip.

### TCGA data analyses

The analysis of TCGA miR-182 expression data in breast invasive carcinoma and normal tissues was based upon data generated by the TCGA Research Network: http://cancergenome.nih.gov. For metastasis-free survival analysis, patient clinical information was retrieved from the TCGA database. The median miR-182 expression level of the whole cohort was used as the cutoff to stratify all patients or the patients of luminal or triple negative subtypes into two groups with high or low expression of miR-182. The non-survival event was defined as distant metastasis or death. Survival probability of the two patient groups were compared with Kaplan-Meier curve. The HER2+ subtype was not analysed due the small number of patients.

### Statistical analysis

Two-sided independent student's *t*-test without assumption of equal variance was performed to analyse the results of *in vitro* assays, animal experiments and clinical samples. Error bars in figures represent standard deviation of the experimental repeats. Quantification of western blots and immunohistochemistry analyses were performed with ImageJ. The band intensities of analysed proteins of western blots were normalized to that of GAPDH. The quantification data of western blots and immunohistochemistry can be found in Supplementary Fig. 12. The original scans of immuno-blots can be found in Supplementary Fig. 13.

### Data availability

All relevant data supporting the findings of this study are available within the article, in Supplementary Information, or from the authors on request.

## Additional information

**How to cite this article:** Yu, J. *et al*. MicroRNA-182 targets SMAD7 to potentiate TGFβ-induced epithelial-mesenchymal transition and metastasis of cancer cells. *Nat. Commun.* **7,** 13884 doi: 10.1038/ncomms13884 (2016).

**Publisher's note:** Springer Nature remains neutral with regard to jurisdictional claims in published maps and institutional affiliations.

## Supplementary Material

Supplementary Information Supplementary Figures, Supplementary Tables, and Supplementary References.
