# Supplementary material for: MicroRNA-182 targets SMAD7 to potentiate TGFβ-induced epithelial-mesenchymal transition and metastasis of cancer cells
Source: Nat Commun. 2016 Dec 20;7:13884. doi: 10.1038/ncomms13884 (PMC5187443; doi:10.1038/ncomms13884)
Supplement: Supplementary Information — Supplementary Figures, Supplementary Tables, and Supplementary References. [file ncomms13884-s1.pdf]

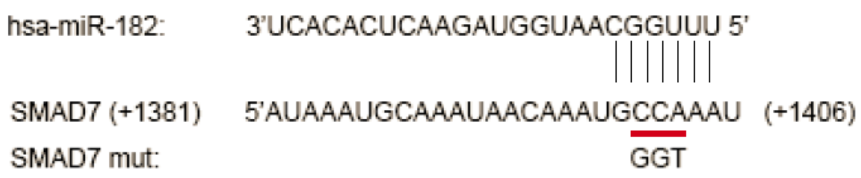

Supplementary Figure 1. The miR-182 binding site of SMAD7 3'UTR and the mutated sequence.

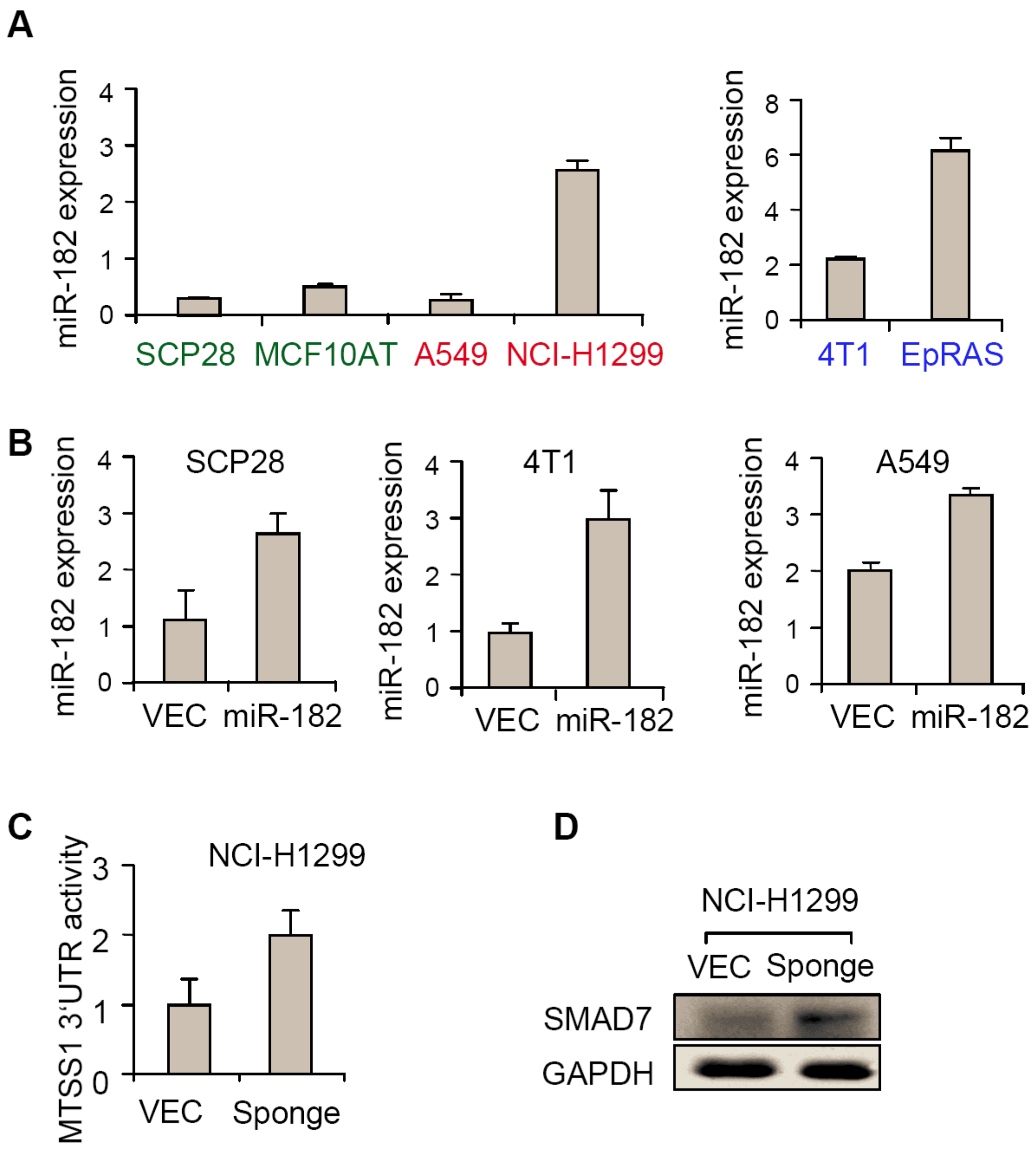

Supplementary Figure 2. Expression of miR-182 and SMAD7 in various cell lines. (**A**) Basal levels of miR-182 expression of the panel of human breast cancer cells (green), human lung cancer cells (red), and murine breast cancer cells (blue) (n=3). (**B**) Validation of miR-182 overexpression in SCP28, 4T1 and A549 (n=3). (**C**) Validation of miR-182 sponge inhibition in NCI-H1299 with a *MTSS1* 3'UTR luciferase reporter[1] (n=4). (**D**) SMAD7 expression in NCI-H1299 treated with the miR-182 sponge. Error bars are defined as s.d.

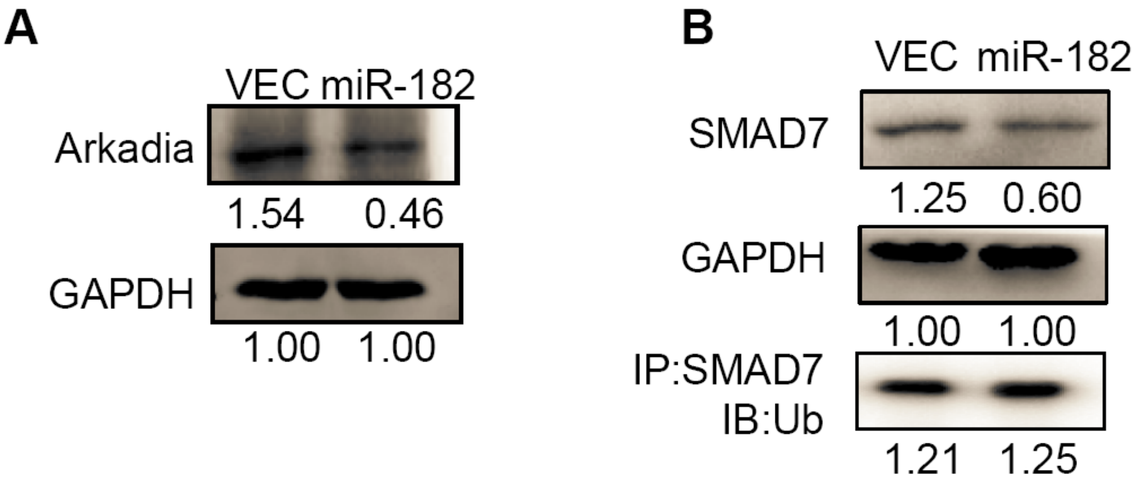

Supplementary Figure 3. miR-182 does not regulate SMAD7 ubiquitination. (**A**) The expression level of Arkadia (RFN111), the SMAD7 E3 ligase after miR-182 overexpression in SCP28. (**B**) SMAD7 ubiquitination status after miR-182 overexpression in SCP28. The cells were treated with the proteosome inhibitor MG-132, and SMAD7 was immunoprecipitated with the anti-SMAD7 antibody followed by anti-Ub detection. The total level of SMAD7 was also shown.

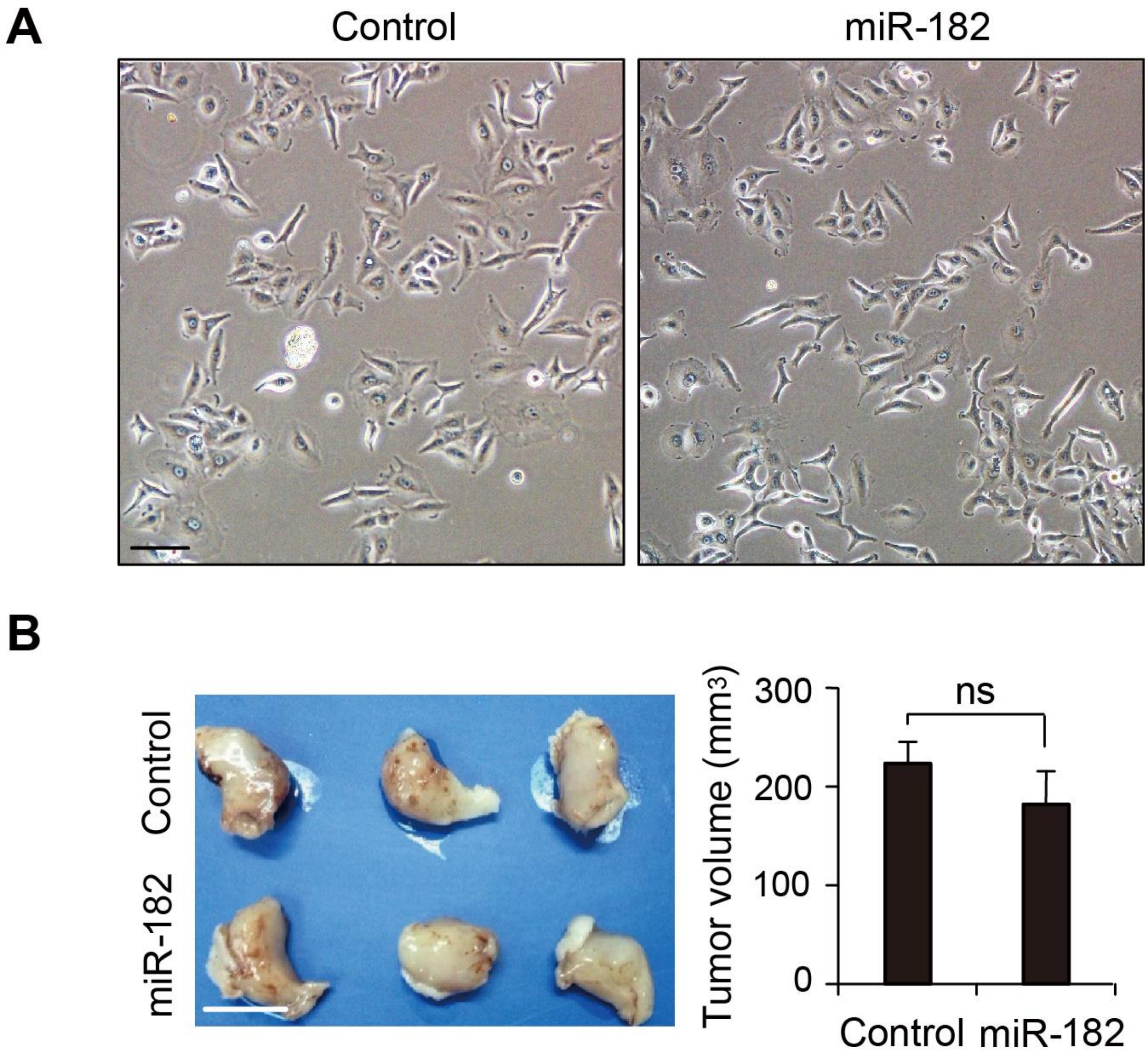

Supplementary Figure 4. miR-182 overexpression does not regulate cancer cell morphology or primary tumor growth. **(A)** Morphological change of A549 after overexpression miR-182. **(B)** Primary tumor growth of 4T1 cells with miR-182 overexpression (n=10). Scale bars, 100 μm (**A**) and 1 cm (**B**). ns, not significant by student's t-test. Error bars are defined as s.d.

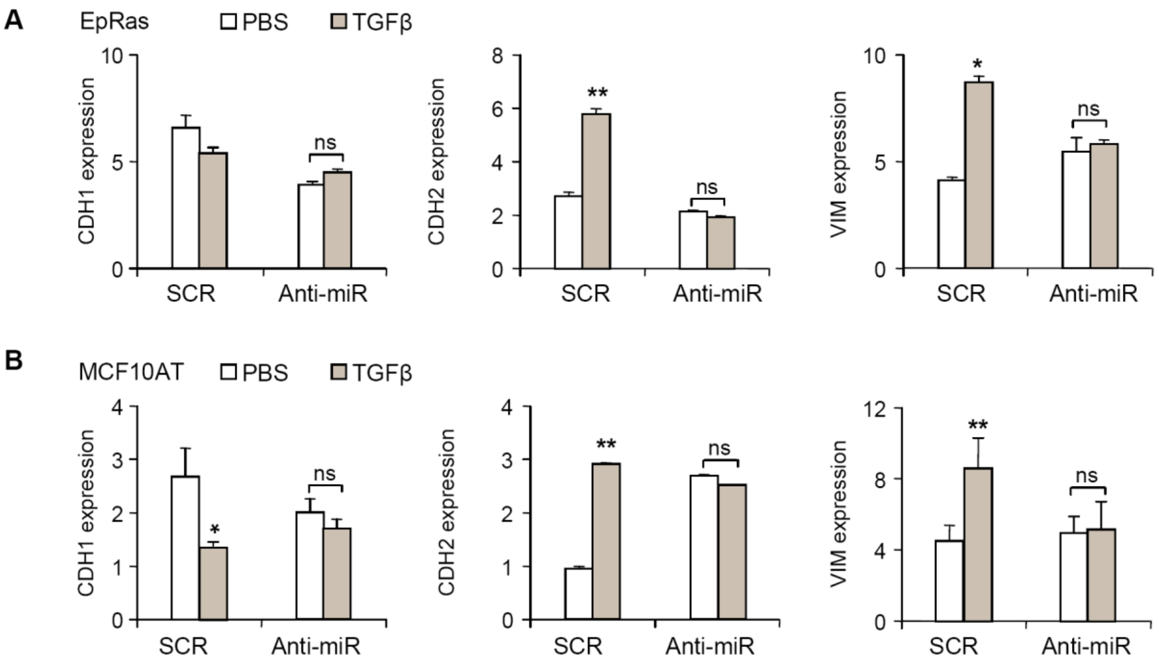

Supplementary Figure 5. EMT marker qPCR analyses after TGFβ treatment and miR-182 inhibition in EpRas (**A**) and MCF10AT (**B**) (n=3). * $P < 0.05$, ** $P < 0.01$ versus control; ns, not significant by student's t-test. Error bars are defined as s.d.

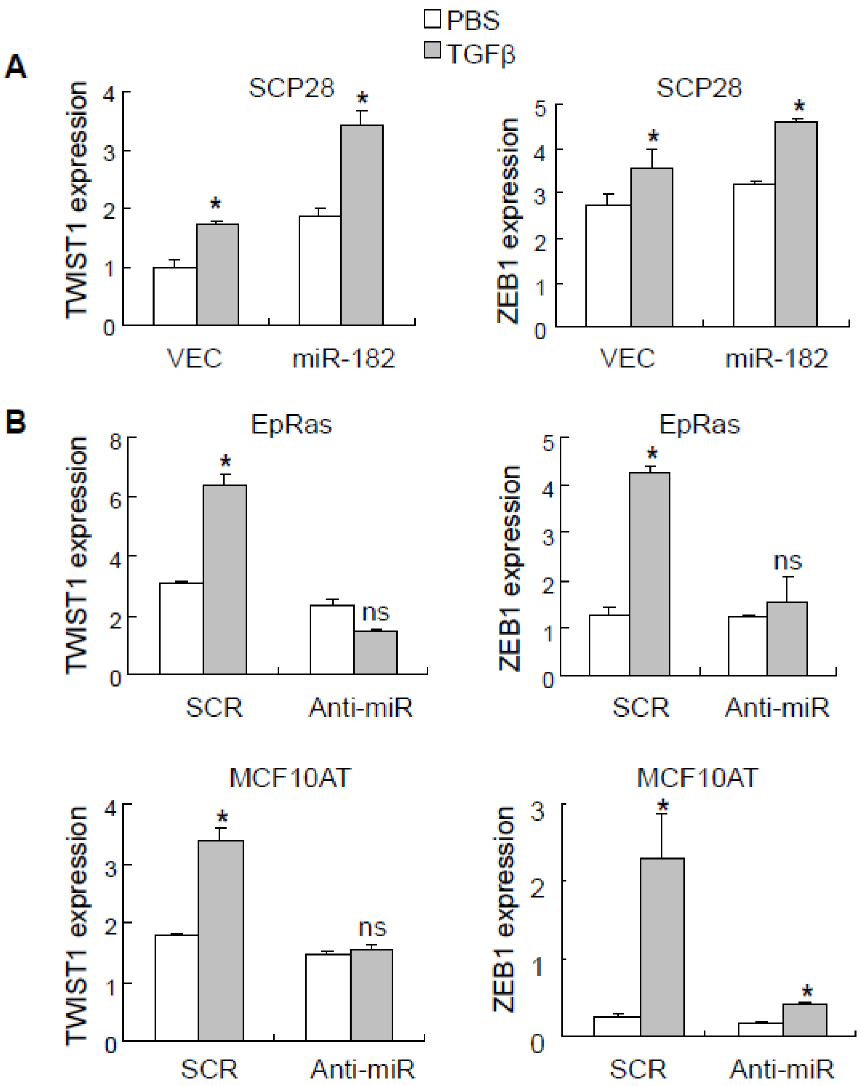

Supplementary Figure 6. TWIST1 and ZEB1 expression analyses after TGFβ treatment, together with miR-182 overexpression in SCP28 (**A**), or with miR-182 inhibition in EpRas and MCF10AT (**B**) (n=3). SCR, scrambled miR-182 inhibitor control. * $P < 0.05$, versus control; ns, not significant by student's t-test. Error bars are defined as s.d.

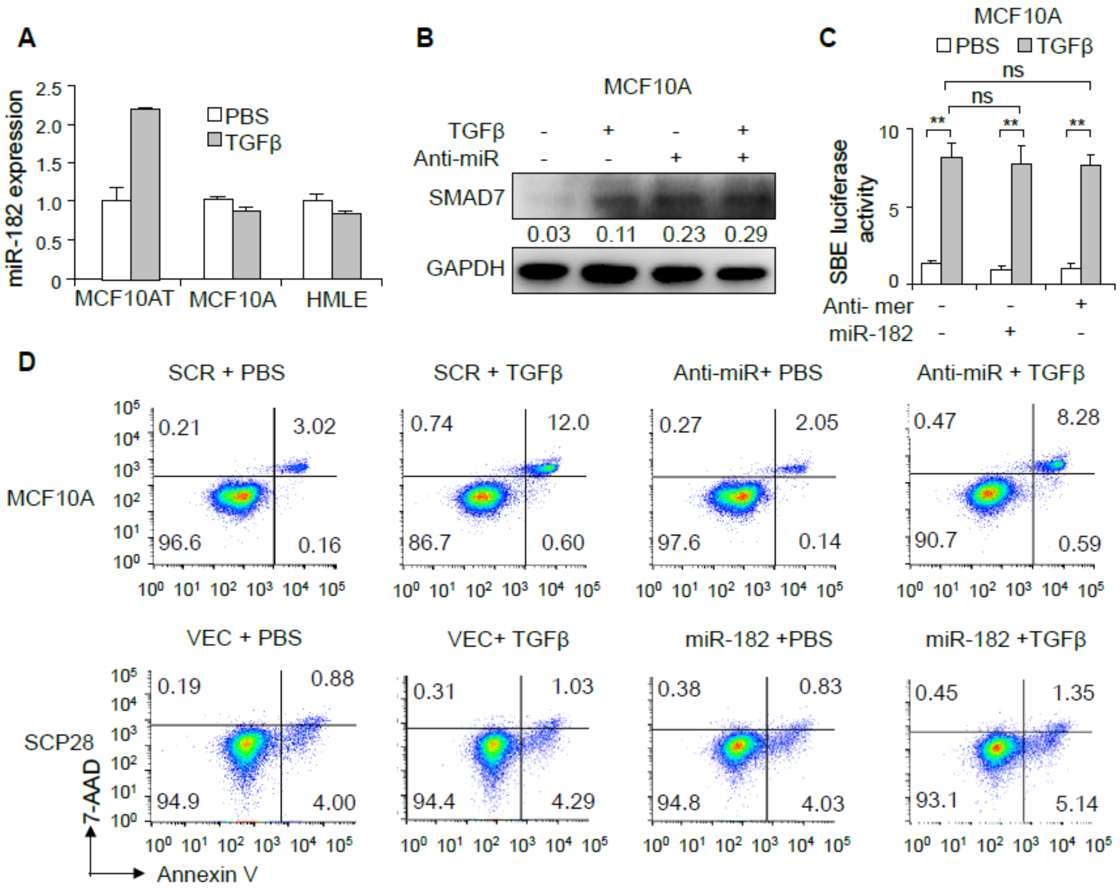

Supplementary Figure 7. The role of miR-182 in TGFβ responses of normal mammary epithelial cells and cancer cells. (**A**) miR-182 mRNA expression in MCF10A and HMLE treated with TGFβ (n=3). (**B**) SMAD7 protein expression in MCF10A after TGFβ stimulation and miR-182 inhibition. (**C**) SBE luciferase reporter assays in MCF10A after TGFβ stimulation and miR-182 overexpression/inhibition (n=4). (**D**) Apoptosis analyses of MCF10A and SCP28 with miR-182 inhibition or overexpression. Numbers denotes the percentage of each subpopulation. ** $P < 0.01$ versus control; ns, not significant by student's t-test. Error bars are defined as s.d.

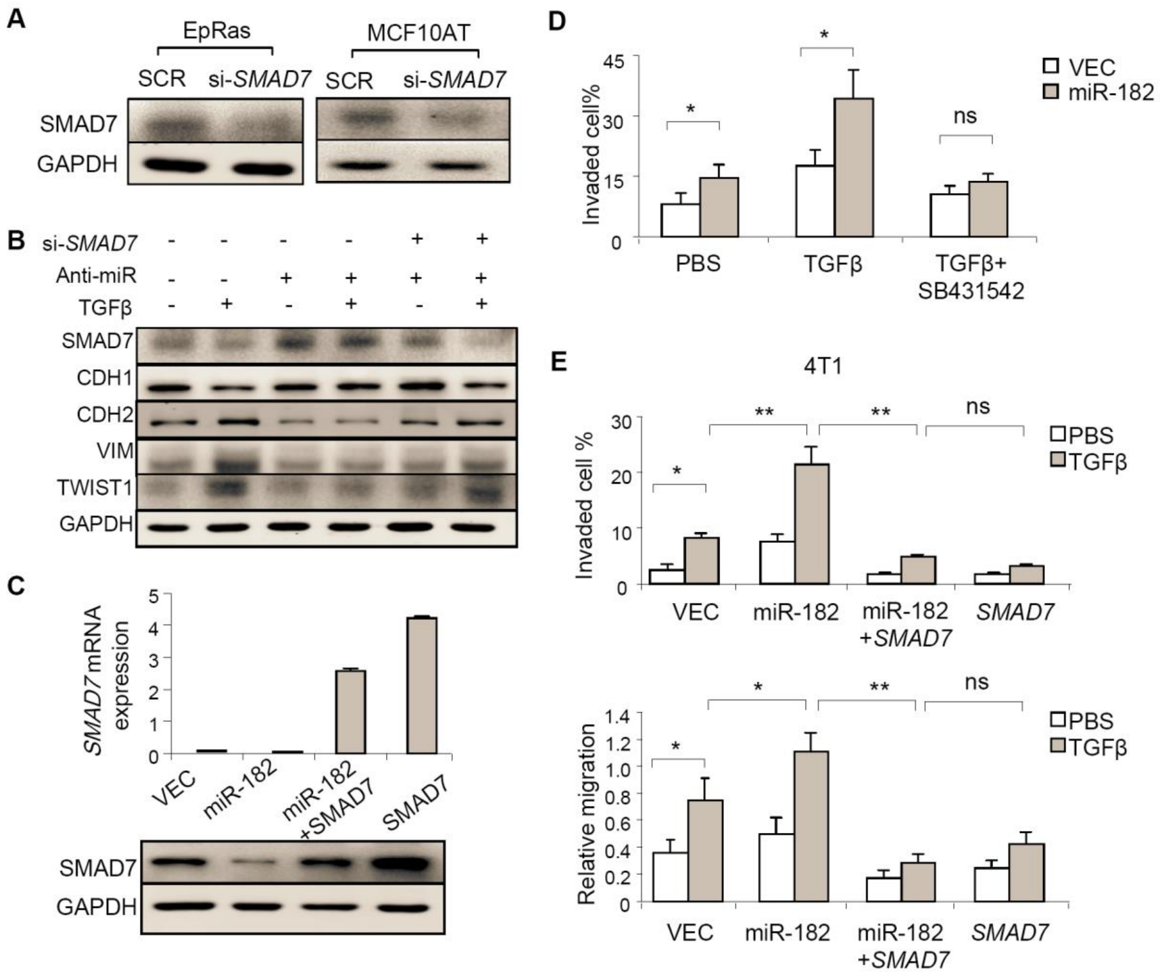

Supplementary Figure 8. SMAD7 mediates the role of miR-182 in EMT and cell invasiveness. (**A**) The efficiency of *SMAD7* siRNA in EpRas and MCF10AT. (**B**) Protein levels of EMT markers and SMAD7 in MCF10AT with TGFβ stimulation, miR-182 inhibition and *SMAD7* silencing. (**C**) Validation of *SMAD7* overexpression in SCP28 cells with miR-182 overexpression. (**D**) Transwell invasion assay of miR-182-overexpressing SCP28 cells with treatment of TGFβ and the TGFβ inhibitor SB431542 (n=4). (**E**) Transwell invasion and migration assays of 4T1 cells treated with TGFβ, and miR-182 and *SMAD7* overexpression (n=4). * $P < 0.05$, ** $P < 0.01$, *** $P < 0.001$; ns, not significant by student's t-test. Error bars are defined as s.d.

.

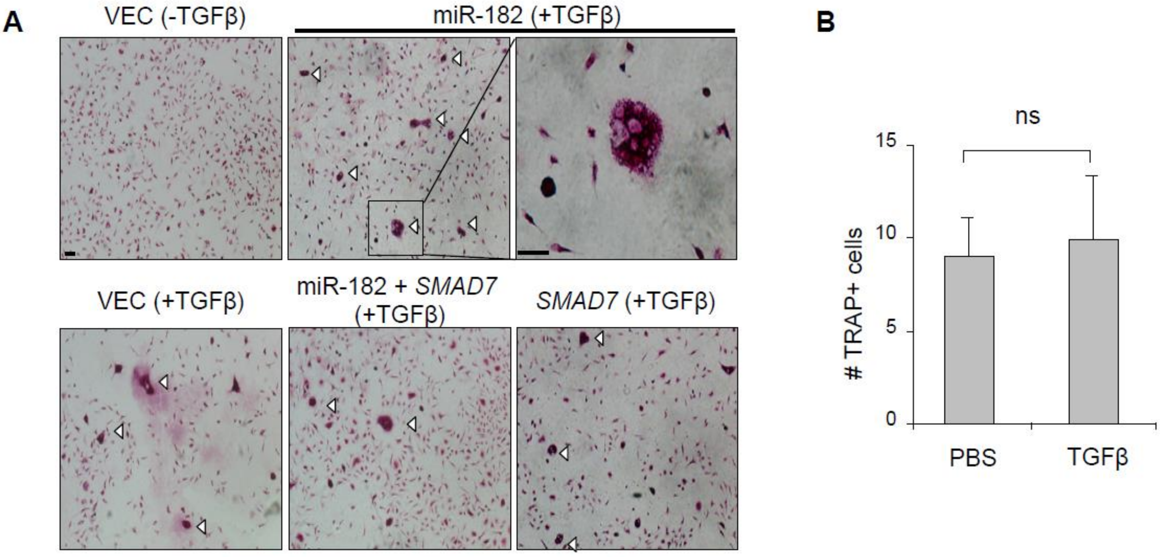

Supplementary Figure 9. SMAD7 mediates the role of miR-182 in osteoclastogenesis. (**A**) Representative TRAP images of osteoclastogenesis from primary mouse bone marrow culturing in conditioned medium of SCP28 with miR-182 and *SMAD7* overexpression. Arrowheads point to multinucleated mature osteoclasts. Scale bars, 50 μm. (**B**) Direct treatment of bone marrow with TGFβ (20 ng/mL) doesn't enhance osteoclastogenesis (n=4). TGFβ was added directly into the bone marrow culture, instead of being added into cancer cell culture prior to condition medium harvest. ns, not significant by student's t-test. Error bars are defined as s.d.

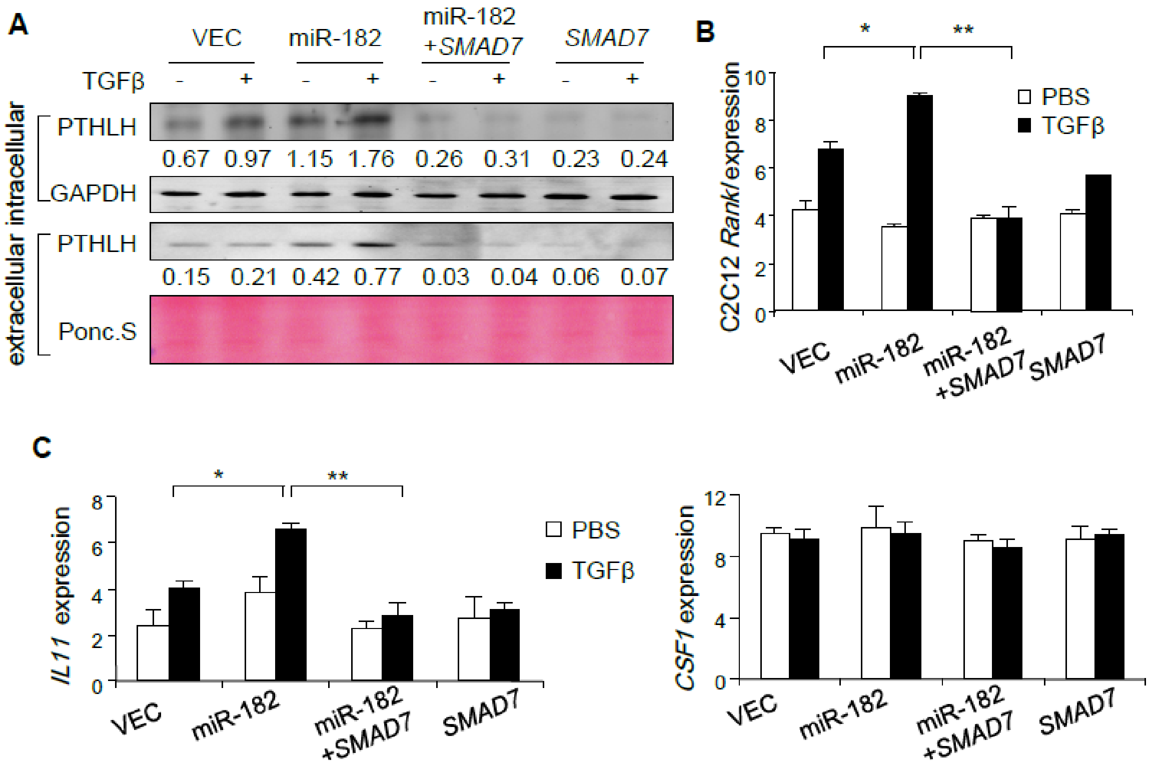

Supplementary Figure 10. The PTHLH-RANKL axis mediates the role of miR-182 in osteoclastogenesis. (**A**) PTHLH protein expression and secretion in SCP28 cells treated with TGFβ. PTHLH quantitation normalized to control was shown. (**B**) *Rankl* expression of C2C12 preosteoblasts cultured in CM of SCP28 cells with miR-182 and/or SMAD7 overexpression and treated with TGFβ (n=3). (**C**) *IL11* and *CSF1* expression in SCP28 cells treated with TGFβ (n=3). * $P < 0.05$, ** $P < 0.01$ by student's t-test. Error bars are defined as s.d.

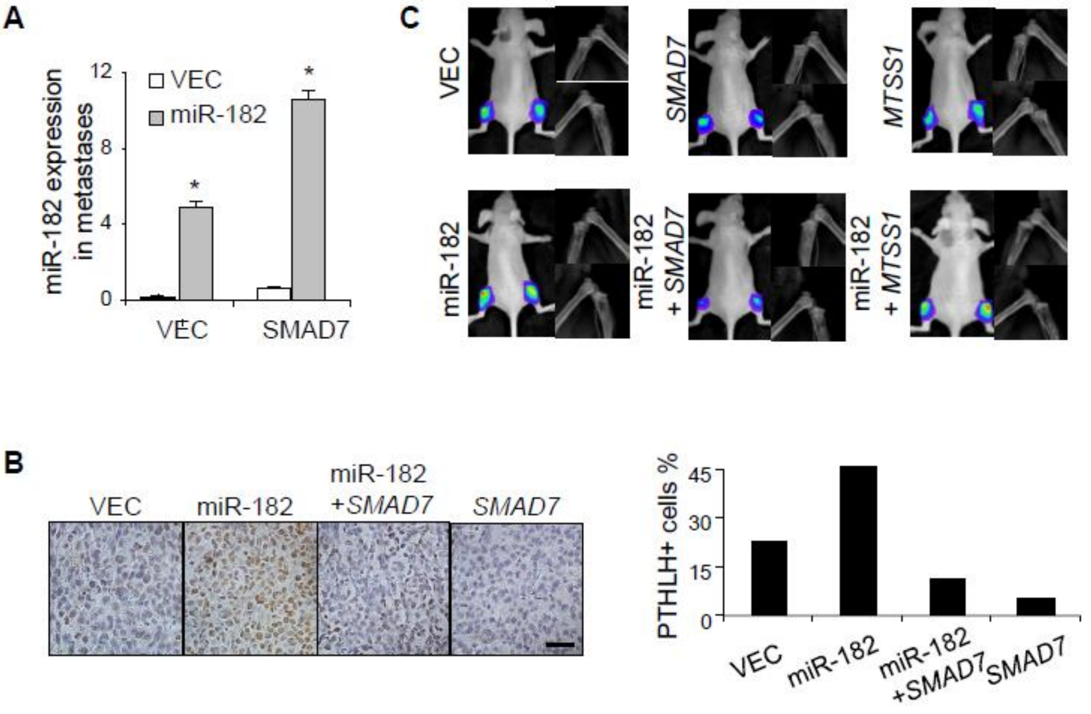

Supplementary Figure 11. SMAD7 rescued miR-182's effects in TGFβ-induced bone colonization. (**A**) miR-182 expression in the bone metastasis tumors caused by SCP28 cells stably transfected with miR-182- and *SMAD7*-expressing vectors (n=3). (**B**) PTHLH IHC analyses of bone metastases by SCP28. Representative and quantitation were shown. (**C**) BLI imaging and X-ray analyses of bone metastases caused by intratibial injection of SCP28 cells with miR-182, *SMAD7* and *MTSS1* overexpression. Scale bars, 100 μm. * $P < 0.05$ versus control by student's t-test. Error bars are defined as s.d.

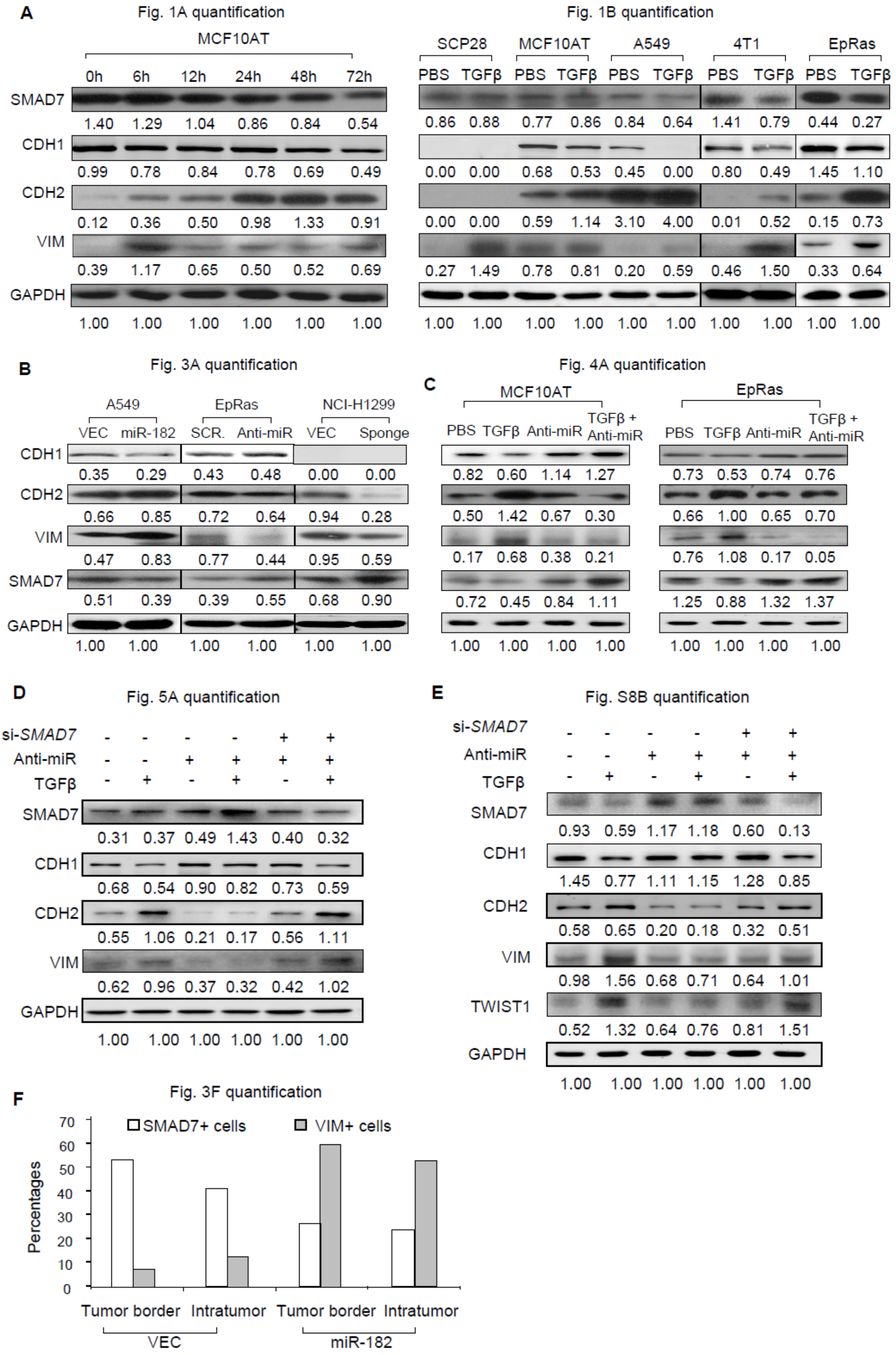

Supplementary Figure 12. ImageJ quantification of the western blots (**A-E**) and immunohistochemistry analysis (**F**) of this study.

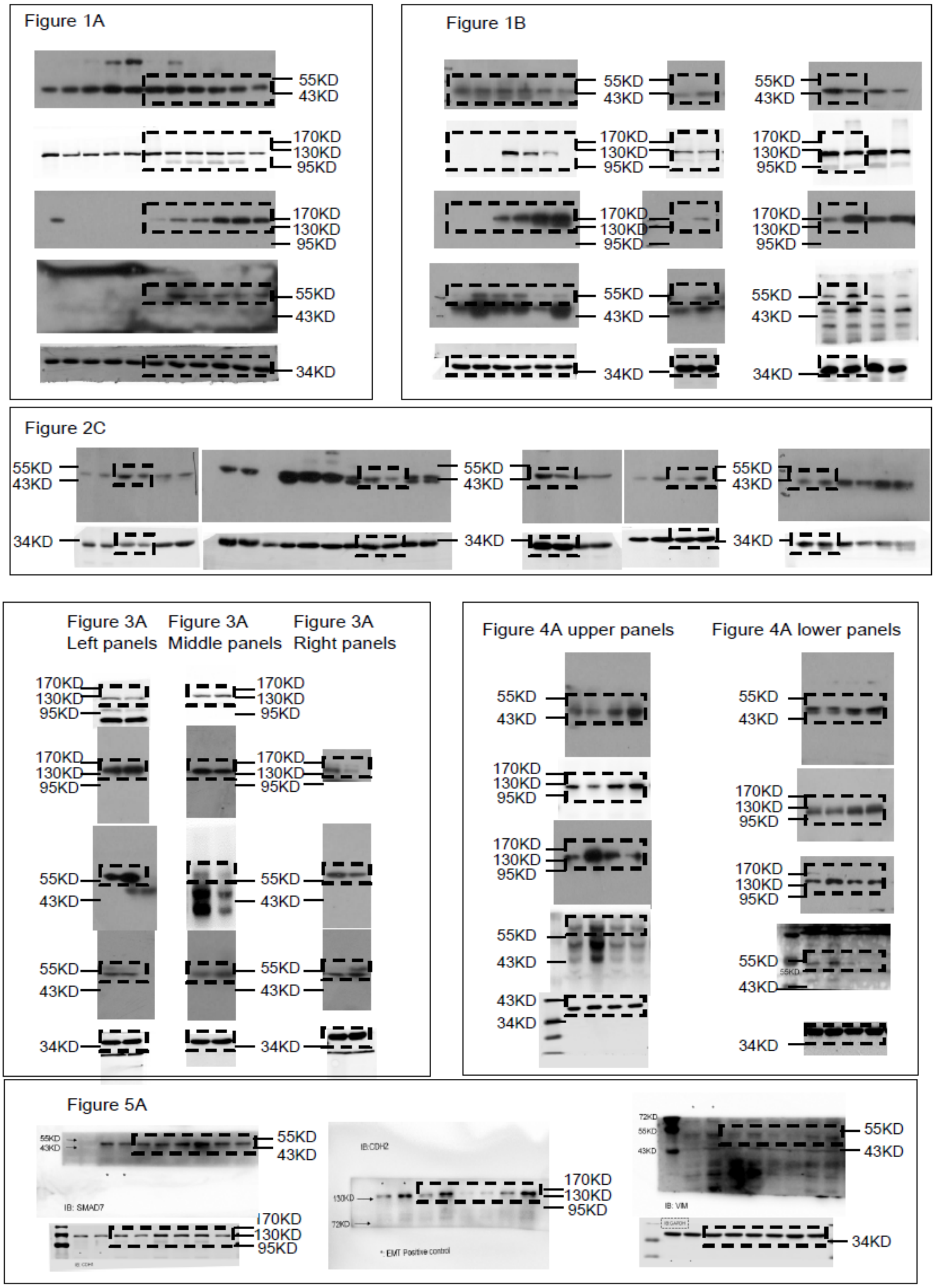

Supplementary Figure 13. Original scans of immuno-blots in the main figures.

Supplementary Table 1: miRNAs predicted to target SMAD7.

miRNA	Positions of the binding sites
miR-15ab/16/195/424/497	55-62
miR-503	56-62
miR-21/590-5p	1122-1129
miR-216a	1230-1236
miR-25/32/92ab/363/367	1329-1335
miR-17/20ab/93/106ab/519d	1366-1372
**miR-182**	1399-1405
miR-181abcd/4262	1461-1467
miR-33a/miR-33b	1384-1390
miR-96/507/1271	1399-1405

Supplementary References:

1 Lei, R. *et al.* Suppression of MIM by microRNA-182 activates RhoA and promotes breast cancer metastasis. *Oncogene* **33**, 1287-1296 (2014).
